# Porous single-crystalline AuPt@Pt bimetallic nanocrystals with high mass electrocatalytic activities†
†Electronic supplementary information (ESI) available: Experimental procedures, characterization data for the Au–Pt bimetallic catalysts and the electrochemical data for the ORR tests. See DOI: 10.1039/c6sc00083e


**DOI:** 10.1039/c6sc00083e

**Published:** 2016-03-14

**Authors:** Lei Zhang, Shengnan Yu, Jijie Zhang, Jinlong Gong

**Affiliations:** a Key Laboratory for Green Chemical Technology of Ministry of Education , School of Chemical Engineering and Technology , Tianjin University , Collaborative Innovation Center of Chemical Science and Engineering , Tianjin 300072 , China . Email: jlgong@tju.edu.cn

## Abstract

Au–Pt bimetallic structures can effectively improve the activity and stability of catalysts in several fuel cell related electrochemical reactions. However, most of the methods for the preparation of Au–Pt nanocrystals (NCs) with core–shell structures are step-wise syntheses, which are adverse for reducing the production costs and the scale-up process. This paper describes a one-pot synthesis of rhombic dodecahedral AuPt@Pt bimetallic nanocrystals with dendritic branches. The dendritic branches on the surfaces were grown through oriented attachment and the whole particle exhibited a single-crystal structure. The thickness of the dendritic Pt shell can be controlled by tuning the introduced Pt precursor. With the Au-enhancement effect arising from the Au–Pt bimetallic core and high atom utilization efficiency provided by the porous structure, the AuPt@Pt bimetallic NCs exhibited greatly enhanced electrocatalytic properties (*e.g.* oxygen reduction reaction and formic acid oxidation) than those of the commercial Pt/C catalyst.

## Abstract 2

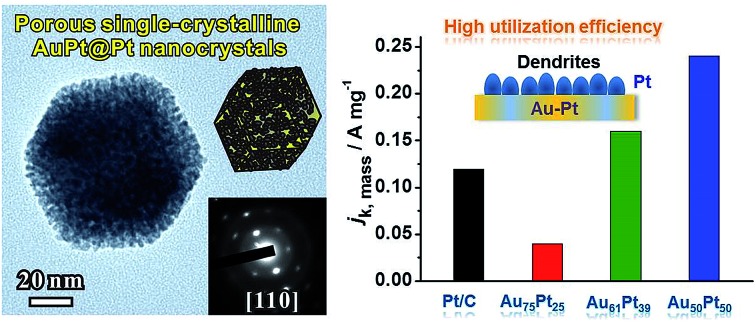
This paper describes the design and synthesis of porous single-crystalline AuPt@Pt bimetallic nanocrystals with excellent mass activities for the oxygen reduction reaction and formic acid oxidation.

## 1. Introduction

Noble metal nanomaterials have attracted numerous attention due to their wide applications in traditional industrial areas and new energy technologies. Pt, as a noble metal, is a critical component of catalysts that are ubiquitous to the energy, pharmaceutical, petroleum, and automobile industries.^1^–^5^ However, its low abundance, limited supply, and ever increasing price have kept motivating researchers to optimize the use of this metal.

Until now, many efforts have been made to improve the properties of Pt-based catalysts. One effective approach is to synthesize Pt–X bimetallic structures, such as Pt–Au,^6^–^10^ Pt–Pd^11^–^14^ and Pt–Ni^15^–^20^ bimetallic nanocrystals (NCs). With the synergistic effect of two metals, some specific physical and chemical properties would be enhanced. Among various types of Pt-based catalysts, Pt–Au catalysts were found to perform with superior stability in addition to having high activity in both anodic and cathodic electrocatalytic reactions.^6^,^21^ However, most studies have focused on investigation of the activities normalized to the electrochemically active surface area (ECSA). Promoting the mass activity of Pt-based materials is the key point for their large-scale commercialization. Generally, the mass activities of catalysts can be enhanced by increasing the atom utilization efficiency through reducing particle size or preparing some specific morphologies such as hollow or porous structures.^22^–^28^ Pt dendrites have recently received much attention because they have a porous structure with high surface areas and their branches can also provide some high-index active sites. It has been reported that the mass activity of Pd–Pt nanodendrites is 2.5 times than that of Pt/C catalyst and 5.0 times than that of supportless Pt-black catalyst.^28^

It can be predicted that the properties of catalysts can be promoted by rational design of a Au–Pt bimetallic structure containing Pt and Au in two compositions, with high atom utilization efficiency. However, most core-Au–shell-Pt NCs have conventionally been prepared by the seed-growth of Pt dendrites on Au particles with specific shapes. The assistance of pre-formed seeds highly increases production costs and limits the simplification of the scale-up process. Accordingly, it is of great importance to develop an effective route to synthesize Au–Pt dendritic structures in the absence of pre-formed seeds. This paper describes a one-pot synthesis of AuPt@Pt bimetallic NCs by a wet-chemical reduction process. With proper reaction kinetics, NCs with a Au–Pt rhombic dodecahedral main core and highly dendritic Pt shell were obtained. In addition, the cores were composed of a Au and Pt alloy, which approached a lower lattice mismatch for the epitaxial growth of Pt dendritic branches. Therefore, it is favourable for the Pt dendritic branches to grow onto the surfaces through oriented attachment. The whole particle had a single crystalline structure even when the thickness of Pt dendritic branches reached 5 nm. Notably, the as-prepared Au–Pt bimetallic NCs exhibited greatly enhanced mass activities and durability as a result of their unique structural characteristics.

## 2. Results and discussion

**Fig. 1:**
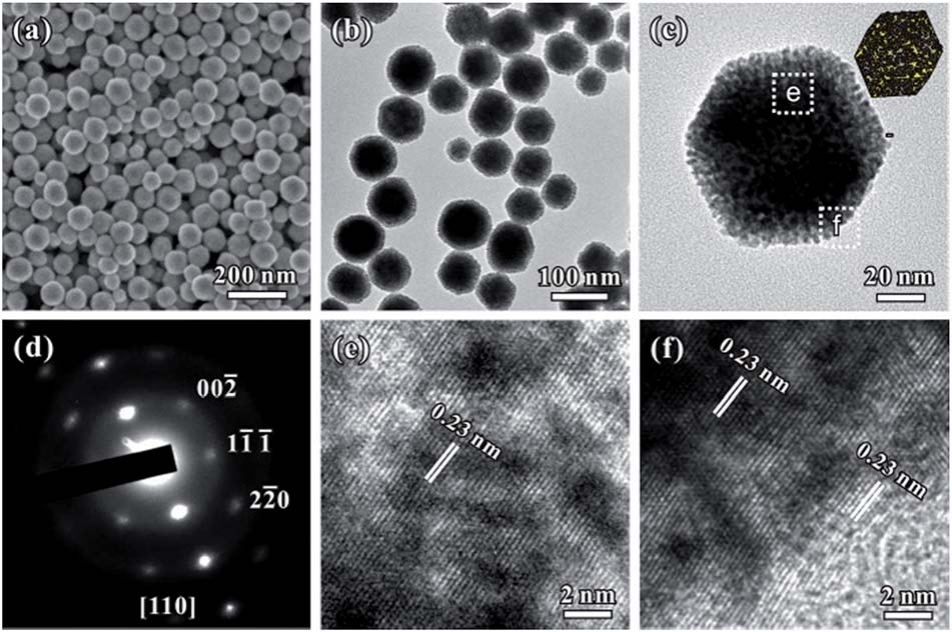
(a and b) SEM and TEM images of the porous rhombic dodecahedral AuPt@Pt bimetallic NCs. (c and d) A TEM image and corresponding SAED pattern of one individual NC. (e and f) HR-TEM images of the two regions of the NC shown in (c).

Fig. 1a and b show typical scanning electron microscopy (SEM) and transmission electron microscopy (TEM) images of the porous rhombic dodecahedral AuPt@Pt bimetallic NCs synthesized using a standard procedure. The bimetallic NCs were prepared *via* a facile one-pot wet-chemical reduction route by using HAuCl_4_ and K_2_PtCl_4_ as metal sources, l-ascorbic acid (AA) as a reducing agent and octadecyl trimethyl ammonium chloride (OTAC) as a capping agent. The SEM image shows that the AuPt@Pt NCs are uniform with an average size of 80 ± 15 nm (Fig. 1a). The TEM image shows that the surfaces of the NCs are composed of numerous dendritic branches instead of smooth facets (Fig. 1b). The average diameter of the dendritic branches is ∼2 ± 0.3 nm. The exposed facets of the branches are expected to be a small fraction of particularly active facets, in addition to some {100} or {110} basic facets.^28^ We have also taken a careful survey of the high-magnification SEM and TEM images of some NCs in different orientations (Fig. S1a and b†). Although the outlines of these NCs are quite different, they can be matched by the same rhombic dodecahedral model projected from the corresponding orientation as shown in the schematic models listed in the right hand column of Fig. S1.† The structural features of the as-prepared rhombic dodecahedral AuPt@Pt bimetallic NCs are further revealed by the TEM image of one individual particle and the corresponding selected area electron diffraction (SAED) pattern as shown in Fig. 1c and d, respectively. The schematic model matches the as-prepared NC well along the [110] direction. More interestingly, the SAED pattern suggests that the particle is a single crystal, indicating that the dendritic branches on the surfaces were grown through highly oriented attachment. The HR-TEM images (Fig. 1e and f) recorded from the region boxed in Fig. 1c reveal a periodic lattice extending across the entire particle and the dendritic branches, suggesting the same orientation on the whole NC. The lattice spacing of 0.23 nm can be assigned to the (111) planes of face-centered cubic (fcc) Au.

**Fig. 2:**
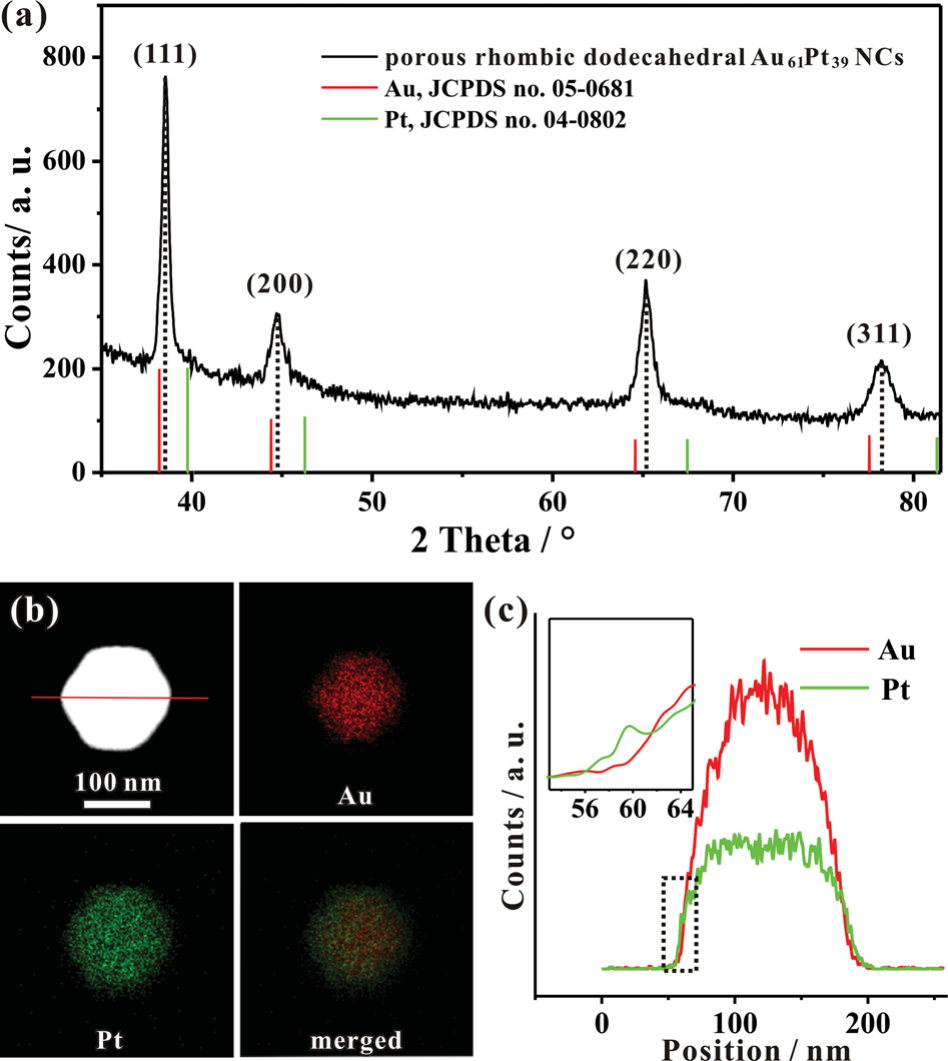
(a) XRD pattern of the typical porous rhombic dodecahedral AuPt@Pt bimetallic NCs. (b) HAADF-STEM image and corresponding HAADF-STEM-EDS elemental mapping of one individual NC. (c) HAADF-STEM-EDS cross-sectional compositional line profile as marked in (b).

We determined the crystal structure of the porous rhombic dodecahedral AuPt@Pt bimetallic NCs by X-ray powder diffraction (XRD). XRD peaks of the as-prepared NCs can be indexed as the fcc structure. As shown in Fig. 2a, the diffraction peaks of the as-prepared porous rhombic dodecahedral NCs appear between the peaks for pure fcc Au and Pt, suggesting the formation of Au–Pt bimetallic NCs. This result is in good agreement with the energy dispersive X-ray spectroscopy (EDS) (Fig. S2†) results collected from the whole particle shown in Fig. 1c. The mole fraction of Pt in the product was determined to be 39% by inductively coupled plasma atomic emission spectroscopy (ICP-AES). More definite compositional distribution information of Au and Pt was provided by high-angle annular dark-field scanning transmission electron microscopy (HAADF-STEM) as well as elemental mapping images shown in Fig. 2b. The results demonstrate that Au and Pt are well overlapped in the middle part. It should be pointed that a thin Pt shell was observed at the outermost surface, which indicates that the dendritic branches of the NCs consist of pure Pt. The cross-sectional compositional line-scanning profile of the individual NC shows that the onset position of the Pt signal is at around 56 nm which is 2 nm smaller than that of Au, indicating a 2 nm thickness of the dendritic Pt shell (Fig. 2c). Accordingly, it can be concluded that the as-prepared porous rhombic dodecahedral NCs are AuPt@Pt bimetallic NCs.

**Fig. 3:**
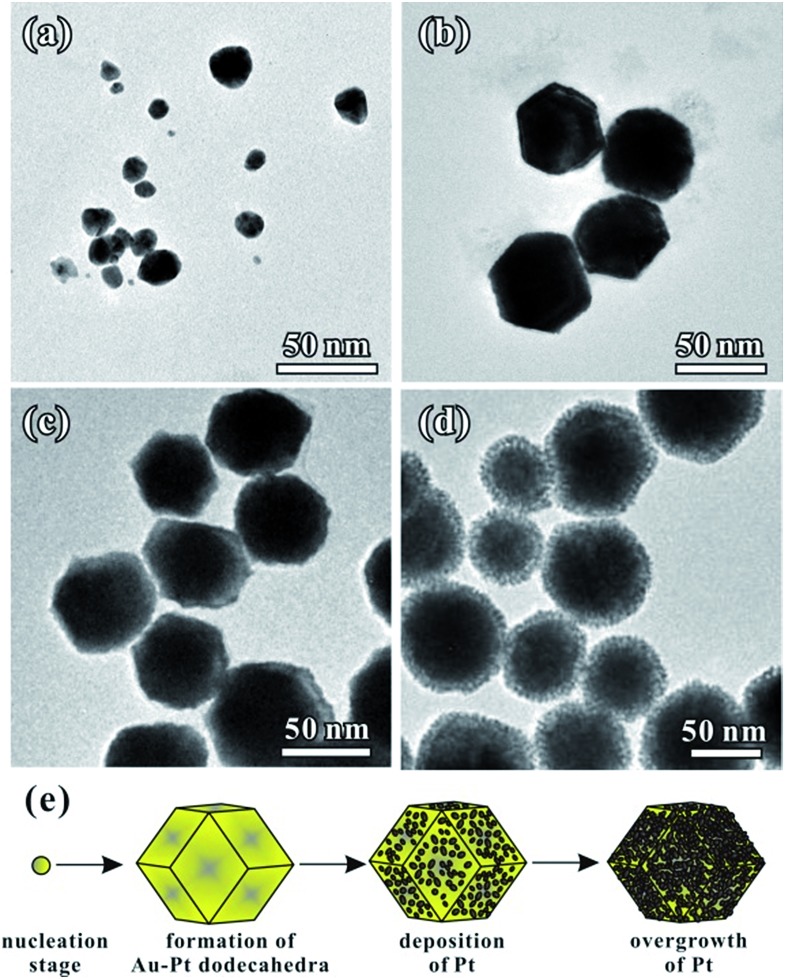
TEM images of the typical porous rhombic dodecahedral AuPt@Pt bimetallic NCs obtained using the standard procedure at different reaction times, (a) 1 min, (b) 5 min, (c) 10 min and (d) 1 h, showing the evolution of morphology with time. (e) A schematic illustration showing the formation process of the Au–Pt bimetallic NCs.

We performed time-dependent reaction experiments to unravel the growth mechanism of the Au–Pt NCs. Fig. 3a–d show the morphologies of Au–Pt bimetallic NCs at different stages of the standard synthesis. As shown in Fig. 3a, the products formed at *t* = 1 min were particles with a size of 20 ± 5 nm. The morphology of the NCs did not exhibit a rhombic dodecahedral shape at the early stage of the growth process. As the synthesis proceeded, the irregular particles evolved to uniform rhombic dodecahedral NCs and the average size of the particles was increased to 50 nm at *t* = 5 min (Fig. 3b). It can be observed that the surfaces of the NCs obtained are smooth, indicating that no dendritic branches formed at this stage. When the reaction time was increased to 10 min, the size of obtained NCs increased to almost 80 nm. In addition, the surface of the NCs became rough as shown in Fig. 3c, suggesting that small Pt particles started to orientedly attach on the surface of the rhombic dodecahedral NCs. Finally, when the reaction time was increased to 1 h, the average size of the obtained NCs reached 80 nm (Fig. 3d). The dendritic branches can be clearly observed in the TEM image. The growth was essentially terminated at *t* = 1 h and the dimensions of the Au–Pt NCs did not show any change when the reaction was prolonged. Fig. 3e shows a schematic illustration of the shape evolution of Au–Pt NCs. At the beginning of the reaction, small particles with irregular shapes were formed. The small particles evolved to form rhombic dodecahedral NCs with smooth surfaces with the passage of time, then Pt particles started to grow onto the surfaces of the NCs after *t* = 10 min. Thanks to the formation of a Au–Pt alloy core, a lower lattice mismatch was achieved for the epitaxial growth of Pt dendritic branches. It is favorable for the Pt dendritic branches to grow on the surfaces through oriented attachment. As a result, an increasing number of Pt particles orientedly attached on the surface of the NCs with a prolonged period of time, forming a dendritic branch shell. The shape evolution of the AuPt@Pt NCs is consistent with the UV-vis spectra taken at different times during the reaction (Fig. S3†). At the beginning of the reaction (*t* = 0 min), two peaks at 228 nm and 324 nm in the UV-vis spectra of the solution correspond to AuCl_4_^–^.^29^ At *t* = 5 min, these two peaks disappeared abruptly, indicating that most of the AuCl_4_^–^ was consumed. Another peak could be observed at 248 nm, which was contributed by Pt(ii).^30^ When the reaction proceeded, the peak at 248 nm decreased gradually. In addition, the LSPR peak red-shifted from 619 nm to 713 nm because of the incorporation of Pt, indicating the overgrowth process of Pt onto the Au particles (Fig. S3b†). With the consumption of the reductant, Pt(ii) could not be reduced to Pt(0) immediately and some of the Pt(ii) converted to [PtCl_4_(OTA)_2_] complexes in the solution. As a result, a peak originating from the [PtCl_4_(CTA)_2_] complexes appeared at around 300 nm.^31^ At *t* = 60 min, the reaction system finally reached a stable balance and the UV-vis spectra did not change any more. The different reactions between Au and Pt precursors can be further proved by HAADF-STEM-EDS analysis at different positions on one individual particle. The branches are confirmed to consist of pure Pt. Furthermore, with the tested area moving from the outer surface to the center of the NC, the Pt atomic ratio decreased from 100% to 16.1% (Fig. S4†). Based on the above analysis, the whole growth process of AuPt@Pt NCs can be described as two stages: (1) the formation of Au–Pt rhombic dodecahedra and (2) the overgrowth of Pt dendritic branches on the rhombic dodecahedra.

To examine how other parameters would influence the formation of rhombic dodecahedral AuPt@Pt NCs, a set of control experiments based on the standard procedure were conducted. As shown in Fig. S5a,† irregular porous Au–Pt bimetallic particles were obtained when the reaction was carried out at room temperature. In comparison, dodecahedral Au–Pt bimetallic NCs could still be obtained when the reaction temperature was increased to 90 °C (Fig. S5b†). However, the size of the particles became nonuniform. These results could be related to the difference in reduction kinetics caused by various temperatures. At room temperature the precursors were more difficult to reduce, resulting in the formation of attached small particles, whereas the nonuniform products obtained at higher temperature were caused by the heterogeneous growth of particles. These results indicate that proper reduction kinetics is critically important for the formation of rhombic dodecahedral Au–Pt bimetallic NCs. We also carried out a control experiment to elucidate the role of OTAC in the synthesis. First, we used cetyltrimethyl trimethyl ammonium chloride (CTAC) in the same molar amount as OTAC to investigate the influence of the chain length of surfactant. We obtained rhombic dodecahedral Au–Pt bimetallic NCs with nonuniform size as shown in Fig. S5c.† In this case, a relatively weak protective capacity of CTAC induced a faster reaction kinetics, which had a similar influence as a higher temperature. When octadecyl trimethyl ammonium bromide (OTAB) was added into the standard synthesis of Au–Pt bimetallic NCs, some of the products exhibited a cubic shape due to the strong absorption stability of Br^–^ with Au (100) surfaces (Fig. S5d†).^32^ These results indicate that the selection of a surfactant with a proper protective capacity and anion is also important for the formation of rhombic dodecahedral Au–Pt bimetallic NCs.

**Fig. 4:**
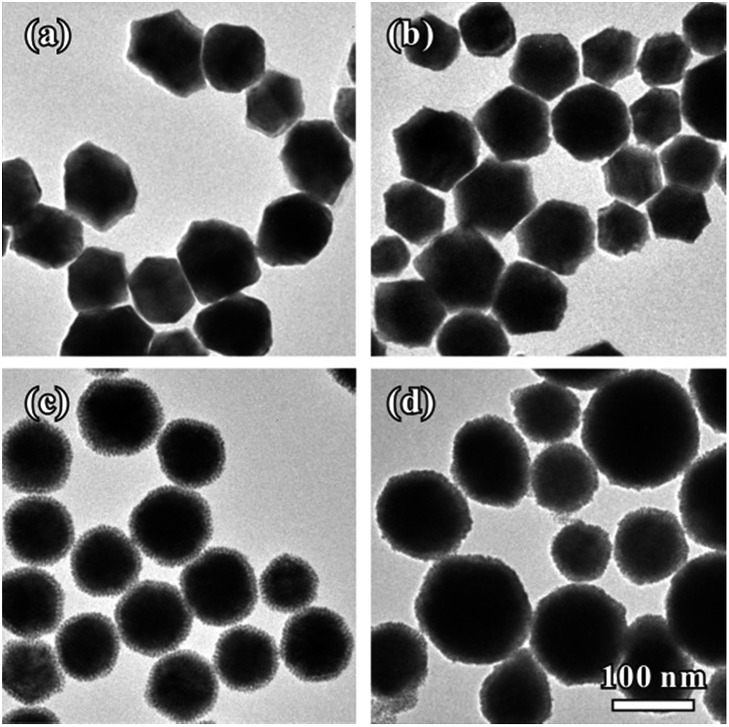
TEM images of the Au–Pt bimetallic NCs that were prepared using the standard procedure, except for variation in the introduced amount of K_2_PtCl_4_ (1 mM): (a) 0.5 mL, (b) 1.0 mL, (c) 2.0 mL and (d) 3.0 mL.

Interestingly, we found that the thickness of Pt dendritic branches on Au–Pt NCs can be easily tuned by changing the introduced amount of Pt source. Fig. 4 shows the formation of Au–Pt bimetallic NCs with different morphologies as the amount of K_2_PtCl_4_ added into the reaction solution was changed. When we introduced a small amount of K_2_PtCl_4_ at a Pt/Au molar ratio of 1 : 6, the products were uniform rhombic dodecahedral Au–Pt NCs with smooth surfaces (Fig. 4a). In this case, the Pt source was simultaneously consumed with AuCl_4_^–^ in the first stage of the whole growth process. When the amount of K_2_PtCl_4_ was increased to a Pt/Au molar ratio of 1 : 3, we obtained rhombic dodecahedral Au–Pt NCs with several particles attached on the surface (Fig. 4b). The surfaces of the obtained NCs are rough as shown in Fig. S6a–c.† The whole particle is still a single crystal according to SAED analysis (Fig. S6d†). The HR-TEM images clearly show that small particles were orientedly attached on the surface of the rhombic dodecahedra (Fig. S6e and f†). Elemental mapping (Fig. S7a†) obtained by HAADF-STEM-EDS reveals a homogenous distribution of Au and Pt in the individual particle, while the compositional line-scanning profiles across the single nanocrystal (Fig. S7b†) show a rather homogenous distribution of both Au and Pt in each nanocrystal. When the amount of K_2_PtCl_4_ was increased to a Pt/Au molar ratio of 2 : 3, we obtained Au–Pt NCs which were prepared using the typical synthesis (Fig. 1b and 4c). When we further increased the amount of K_2_PtCl_4_ to reach a Pt/Au molar ratio of 1 : 1, the dendritic branches on the NCs became much thicker than those of the Au–Pt synthesized using the standard method. A whole particle had a spherical shape due to its thick dendritic branch shell, as shown in Fig. S8a and b.† The structure of the products is still a single crystal as shown by the SAED pattern (Fig. S8c†) and the thick dendritic branches were also orientedly attached on the surface of the rhombic dodecahedra (Fig. S8d†). Fig. S9a† shows the HAADF-STEM and elemental mapping image of three NCs. The Pt shell can be clearly observed at the outermost surface. Moreover, the cross-sectional compositional line-scanning profile of one individual NC shows that the onset position of the Pt signal is at around 55 nm which is 5 nm smaller than that of Au, indicating that the thickness of the dendritic Pt shell is much thicker than that of AuPt@Pt NCs synthesized using the standard method (Fig. S9b†).

The structures of the three types of samples (shown in Fig. 4a, b and d) were further confirmed through XRD patterns. The XRD peaks of the three NCs can all be indexed to the fcc structure. Every diffraction peak appears between the corresponding peak positions of pure fcc-structured Au and Pt (Fig. S10†), suggesting the successful formation of Au–Pt bimetallic NCs. Furthermore, the peak location position moves to a higher angle when the amount of PtCl_4_^2–^ is increased, indicating an increasing trend in incorporated Pt in the NCs. To determine the precise amount of each component in the Au–Pt bimetallic NCs, the three samples with different thicknesses of Pt shells (shown in Fig. 4a, b and d) were also analyzed by ICP-AES. With the increase of the introduced amount of K_2_PtCl_4_, the mole fractions of Pt in the NCs were 13%, 25% and 50%, respectively.

Since the porous rhombic dodecahedral AuPt@Pt NCs have highly accessible atoms on the porous surfaces, they are expected to be excellent catalysts for various types of reaction. We used the oxygen reduction reaction (ORR) as a model system to characterize the catalytic activities of the Au–Pt catalysts. Cyclic voltammograms (CV) were recorded in 0.10 M aqueous HClO_4_ at a scanning rate of 50 mV s^–1^. First, we obtained the CV curves of Au_75_Pt_25_, Au_61_Pt_39_ and Au_50_Pt_50_ catalysts in a potential region from 0.08 V to 1.78 V (*vs.* reversible hydrogen electrode, RHE) to show the surface coverage of Pt. The CV curves were normalized by the charges responsible for hydrogen desorption. The shoulder peaks at 0.9 V and 1.5 V correspond to O absorption on the Pt and Au surfaces, respectively (Fig. S11†).^33^,^34^ When the Pt content increases, the shoulder peak at 0.9 V appears gradually, while the peak at 1.5 V disappears. This result indicates that the surface was totally covered by Pt atoms when the Pt/Au atomic ratio reached 1 : 1. ECSAs were determined by the charges responsible for hydrogen desorption as shown in Fig. S12a.† The specific ECSAs were calculated to be 27.3, 37.5, 43.3 and 66.0 m^2^ g_Pt_^–1^ for Au_75_Pt_25_, Au_61_Pt_39_, Au_50_Pt_50_, and commercial Pt/C catalysts, respectively. With a relatively thicker porous Pt shell, the ECSA of the Au_50_Pt_50_ catalyst was comparable to that of the Pt/C catalyst even though its size was much larger than that of Pt/C.

**Fig. 5:**
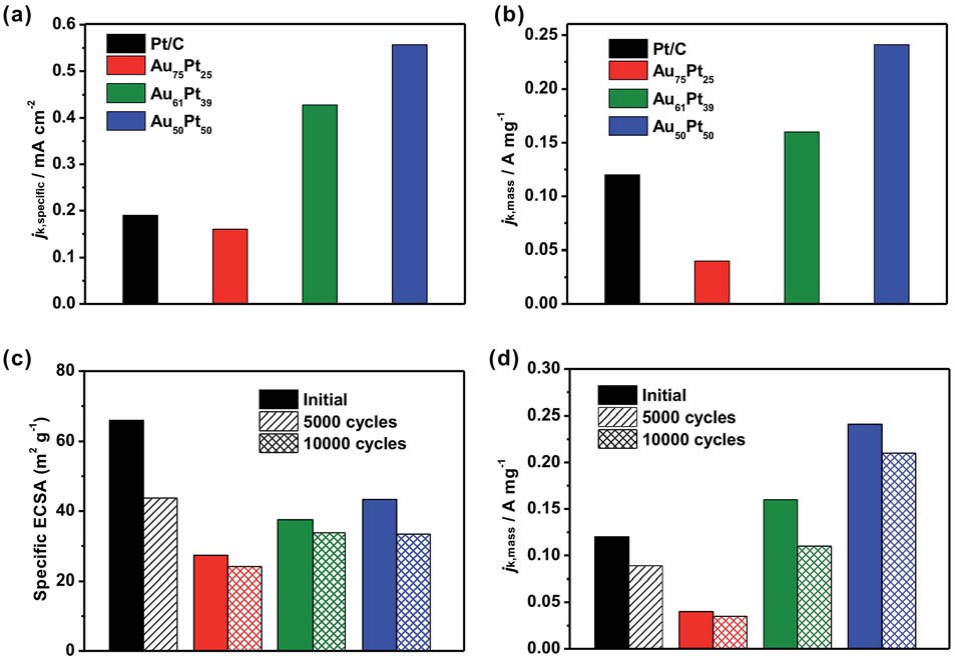
Oxygen reduction performances of three types of AuPt@Pt bimetallic catalysts with different thicknesses of Pt dendritic branches relative to a commercial Pt/C catalyst. (a) Specific and (b) mass activities of the catalysts at 0.9 V_RHE_. (c) Specific ECSAs and (d) mass activities (at 0.9 V_RHE_) of the catalysts before and after accelerated durability testing. The colour scheme applies to all panels.

We then evaluated the electrocatalytic performance of the three types of Au–Pt catalysts for the ORR (Fig. S12b–d†). In order to compare the activity for different catalysts, we normalized the kinetic current to the ECSA (*j*_k,specific_) and Pt mass (*j*_k,mass_), respectively. As shown in Fig. 5a, the Au_61_Pt_39_ and Au_50_Pt_50_ catalysts exhibit greatly improved activity, with *j*_k,specific_ values of 0.43 and 0.55 mA cm^–2^ based on the ECSA at 0.9 V *vs.* RHE, which are 2.3 and 2.9 times greater than that of the Pt/C catalyst (0.19 mA cm^–2^), respectively. The Au_75_Pt_25_ catalyst exhibited worse activity than the Pt/C catalyst, as the Pt/C might contain more active sites, which were provided by the edge and corner atoms of the small particles. The mass activities of the above catalysts show a similar trend to that of the specific activities. As shown in Fig. 5b, the porous Au_61_Pt_39_ and Au_50_Pt_50_ catalysts had an obviously improved *j*_k,mass_ relative to the Pt/C catalyst. The mass activity of the Au_75_Pt_25_ catalyst was much worse than that of Pt/C, as this sample contains no obvious porous Pt shells. These results indicate that the high atom utilization efficiency provided by the dendrites and core–shell structure greatly improves the performance of the catalysts for the ORR. We also tested the long-term stability of the catalysts through accelerated durability tests. The ECSAs of the Au_75_Pt_25_, Au_61_Pt_39_ and Au_50_Pt_50_ catalysts only decreased by 11.8%, 9.9% and 22.8% after 10 000 cycles, respectively; while the ECSA of the commercial Pt/C catalyst decreased by 32.8% after only 5000 cycles (Fig. 5c). For the mass ORR activities at 0.9 V, the Au_50_Pt_50_ catalyst, which exhibited the best activity towards ORR, only showed a 12.5% loss in mass activity after 10 000 cycles, while the loss for the Pt/C catalyst was 25.8% after 5000 cycles (Fig. 5d). These results indicate that all three types of the Au–Pt bimetallic catalysts exhibited greatly improved durability. The formation of a Au–Pt bimetallic structure maintained the activity of Pt atoms after the long term durability tests.^6^ Additionally, the highly oriented single crystalline structure provided a strong force to pin Pt dendritic branches on the surfaces, thus avoiding aggregation (attachment between branches) or detachment from the cores during the durability tests.

Except for the use as cathode catalysts for ORR, Pt-based catalysts can also be applied in anodic catalytic reactions. Hence, we also studied the electrocatalytic performance of the AuPt@Pt catalysts for formic acid oxidation. Fig. S13† reveals the activities of the as-prepared Au–Pt NCs with different thicknesses of dendritic branches, corresponding to Au_87_Pt_13_, Au_75_Pt_25_, Au_61_Pt_39_ and Au_50_Pt_50_ catalysts, respectively. The peak current densities for formic acid oxidation in the positive potential scan were measured to be 4.63, 4.41, 3.66, 3.56 and 0.36 mA cm^–2^ for the Au_87_Pt_13_, Au_75_Pt_25_, Au_61_Pt_39_, Au_50_Pt_50_ and commercial Pt/C catalysts, respectively (Fig. S13a†). It was found that the electrocatalytic abilities of the Au–Pt catalysts are all superior to the commercial Pt/C catalyst. The oxidation current density measured on the Au_87_Pt_13_ NCs is almost 12 times that of the commercial Pt/C catalyst. When the ratio of Pt is increased, the peak current densities reduce from 4.63 to 3.56 mA cm^–2^ due to the exposed {110} surface that was covered by Pt dendritic branches with dominant {100} and {111} facets. It should be pointed out that, with the increase of the Pt shell thickness to 5 nm, the catalysts can still exhibit a great activity due to the small ratio of high index active sites. The curves for mass activity (per unit mass of Pt) of the catalysts are shown in Fig. S13b.† The anodic peak mass activities for the Au_87_Pt_13_, Au_75_Pt_25_, Au_61_Pt_39_, Au_50_Pt_50_ and commercial Pt/C catalysts are 0.52, 0.71, 0.92, 0.78 and 0.17 A mg^–1^, respectively. All of the Au–Pt NCs performed better than the commercial Pt/C catalyst, with the mass activity of the Au_61_Pt_39_ NCs being 5.4 times that of the commercial Pt/C catalyst. In addition, the Au_61_Pt_39_ catalysts exhibited better mass activity than Au_50_Pt_50_, suggesting that a thin Pt shell is helpful for the improvement of mass activity.

## 3. Conclusions

In summary, we have demonstrated a facile synthesis of porous single-crystalline rhombic dodecahedral AuPt@Pt bimetallic NCs. Each porous nanoparticle contains two parts: the rhombic dodecahedral main body and orientedly attached dendritic branches. The samples obtained at different time points suggest that the Au–Pt rhombic dodecahedral alloy were formed first, followed by the overgrowth of the dendritic Pt shell. Through SAED and HR-TEM analysis, we confirmed that the single crystalline structure of the core was transferred to the dendritic branches and the whole particle was a single crystal. The thickness of the dendritic branches can be controlled through tuning the introduced amount of Pt precursor. In addition, the structure can be maintained as a single crystal with an increase in the dendritic branches. The successful preparation of the AuPt@Pt NCs is highly dependent on proper reduction kinetics (70 °C) and a surfactant with an appropriate protective capacity (OTAC). Compared with the commercial Pt/C catalyst, the porous Au_61_Pt_39_ and Au_50_Pt_50_ catalysts exhibited substantially enhanced catalytic activity and good stability toward both the oxygen reduction reaction and formic acid oxidation.

## Supplementary Material

Supplementary information

## References

[cit1] Steele B. C. H., Heinzel A. (2001). Nature.

[cit2] Chen J. Y., Lim B., Lee E. P., Xia Y. (2009). Nano Today.

[cit3] Debe M. K. (2012). Nature.

[cit4] Zhou Z., Tian N., Li J., Broadwell I., Sun S. (2011). Chem. Soc. Rev..

[cit5] Chen A. C., Holt-Hindle P. (2010). Chem. Rev..

[cit6] Zhang J., Sasaki K., Sutter E., Adzic R. R. (2007). Science.

[cit7] Zhou S., McIlwrath K., Jackson G., Eichhorn B. (2006). J. Am. Chem. Soc..

[cit8] Kim Y., Hong J. W., Lee Y. W., Kim M., Kim D., Yun W. S., Han S. W. (2010). Angew. Chem..

[cit9] Ataee-Esfahani H., Wang L., Nemoto Y., Yamauchi Y. (2010). Chem. Mater..

[cit10] Wang S., Kristian N., Jiang S., Wang X. (2009). Nanotechnology.

[cit11] Wang L., Nemoto Y., Yamauchi Y. (2011). J. Am. Chem. Soc..

[cit12] Zhang H., Jin M., Liu H., Wang J., Kim M. J., Yang D., Xie Z., Liu J., Xia Y. (2011). ACS Nano.

[cit13] Yin A.-X., Min X.-Q., Zhu W., Wu H.-S., Zhang Y.-W., Yan C.-H. (2012). Chem. Commun..

[cit14] Peng Z., Yang H. (2009). J. Am. Chem. Soc..

[cit15] Stamenkovic V. R., Fowler B., Mun B. S., Wang G., Ross P. N., Lucas C. A., Markovic N. M. (2007). Science.

[cit16] Wu J., Zhang J., Peng Z., Yang S., Wagner F. T., Yang H. (2010). J. Am. Chem. Soc..

[cit17] Zhang J., Yang H., Fang J., Zou S. (2010). Nano Lett..

[cit18] Cui C., Gan L., Li H. H., Yu S. H., Heggen M., Strasser P. (2012). Nano Lett..

[cit19] Carpenter M. K., Moylan T. E., Kukreja R. S., Atwan M. H., Tessema M. M. (2012). J. Am. Chem. Soc..

[cit20] Choi S.-I., Xie S., Shao M., Odell J. H., Lu N., Peng H.-C., Protsailo L., Guerrero S., Park J., Xia X., Wang J., Kim M. J., Xia Y. (2013). Nano Lett..

[cit21] Zeng J., Yang J., Lee J. Y., Zhou W. (2006). J. Phys. Chem. B.

[cit22] Xu Y., Zhang B. (2014). Chem. Soc. Rev..

[cit23] Teng X., Liang X., Maksimuk S., Yang H. (2006). Small.

[cit24] Zhang L., Roling L. T., Wang X., Vara M., Chi M., Liu J., Choi S.-I., Park J., Herron J. A., Xie Z., Mavrikakis M., Xia Y. (2015). Science.

[cit25] Xie S., Choi S.-I., Lu N., Roling L. T., Herron J. A., Zhang L., Park J., Wang J., Kim M. J., Xie Z., Mavrikakis M., Xia Y. (2014). Nano Lett..

[cit26] Park J., Zhang L., Choi S.-I., Roling L. T., Lu N., Herron J. A., Xie S., Wang J., Kim M. J., Mavrikakis M., Xia Y. (2015). ACS Nano.

[cit27] Wang X., Choi S.-I., Roling L. T., Luo M., Ma C., Zhang L., Chi M., Liu J., Xie Z., Herron J. A., Mavrikakis M., Xia Y. (2015). Nat. Commun..

[cit28] Lim B., Jiang M., Camargo P. H. C., Cho E. C., Tao J., Lu X., Zhu Y., Xia Y. (2009). Science.

[cit29] Gangopadhayay A. K., Chakravorty A. (1961). J. Chem. Phys..

[cit30] Herricks T., Chen J., Xia Y. (2004). Nano Lett..

[cit31] Zhang J., Xie S., Kuang Q., Han X., Xie Z., Zheng L. (2011). Chem.–Eur. J..

[cit32] Liu X.-L., Liang S., Nan F., Yang Z.-J., Yu X.-F., Zhou L., Hao Z.-H., Wang Q.-Q. (2013). Nanoscale.

[cit33] Jiang Q., Jiang Z., Zhang L., Lin H., Yang N., Li H., Liu D., Xie Z., Tian Z. (2011). Nano Res..

[cit34] Zhang L., Chen D., Jiang Z., Zhang J., Xie S., Kuang Q., Xie Z., Zheng L. (2012). Nano Res..

