# Supplementary material for: Porous single-crystalline AuPt@Pt bimetallic nanocrystals with high mass electrocatalytic activities
Source: Chem Sci. 2016 Mar 14;7(6):3500–5. doi: 10.1039/c6sc00083e (PMC6007209; doi:10.1039/c6sc00083e)
Supplement: Supplementary file 1 [file SC-007-C6SC00083E-s001.pdf]

Electronic Supplementary Material (ESI) for Chemical Science.
This journal is © The Royal Society of Chemistry 2016

Supporting Information

Porous Single-Crystalline AuPt@Pt Bimetallic Nanocrystals with High Mass Electrocatalytic Activities

Lei Zhang, Shengnan Yu, Jijie Zhang and Jinlong Gong*

Key Laboratory for Green Chemical Technology of Ministry of Education, School of Chemical Engineering and Technology, Tianjin University; Collaborative Innovation Center of Chemical Science and Engineering, Tianjin 300072, China

*Email: jlgong@tju.edu.cn

Experimental Section

Chemicals and Materials. Chloroauric acid hydrated ($HAuCl_4 \cdot 4H_2O$, 99%) and potassium tetrachloroplatinate(II) (K_2PtCl_4, 99%) were purchased from J&K China Chemical Ltd. Octadecyl trimethyl ammonium chloride (OTAC, 99%), octadecyl trimethyl ammonium bromide (OTAB, 99%), cetyltrimethyl trimethyl ammonium chloride (CTAC, 99%) and L-ascorbic acid (AA, analytical grade) were purchased from Sinopharm Chemical Reagent Co. Ltd. (Shanghai, China). The commercial Pt/C (20%) was purchased from Alfa Aesar. All reagents were used as received without further purification. All aqueous solutions were prepared with ultrapure water.

Preparation of porous rhombic dodecahedral AuPt@Pt bimetallic NCs. In a typical procedure for the synthesis of porous rhombic dodecahedral Au-Pt bimetallic NCs, an aqueous K_2PtCl_4 solution (2.0 mL, 1.0 mM) and a OTAC aqueous solution (3.0 mL, 0.01 M) were added into an aqueous $HAuCl_4$ solution (3.0 ml, 1.0 mmol/L) in turn. After homogeneous mixing, the solution was placed in a vial and pre-heated at 70 ^oC in a water bath for 10 min. Subsequently, a freshly prepared, aqueous AA solution (0.20 mL, 0.10 M) was quickly added with a gentle shaking to this solution and left undisturbed for 1 hour at 70 ^oC. After the reaction, the samples were centrifuged at 6000 rpm for 10 min and washed by ultrapure water for three times.

Electrochemical measurements for oxygen reduction reaction (ORR). Electrochemical measurements were conducted using a RDE connected to a CHI 660E potentiostat (CH Instruments). A leak-free Ag/AgCl/NaCl (3M) electrode (BASi) was used as the reference. All potentials were converted to values with reference to the reversible hydrogen electrode (RHE) by adding 0.29 V. The counter electrode was a platinum foil (2×2 cm^2). Firstly, to investigate the surface coverage of Pt, we dripped 20 μl of the ethanol suspensions (0.2 mg_{Pt}/mL) of the as-prepared Au-Pt NCs (without loading) onto the surface of the glassy carbon electrode and dried at 50 ^oC in an oven. The glassy carbon electrode loaded with the as-prepared catalysts was electrochemical cleaned by continuous potential cycling between -0.08 and 1.10 V at 50 $mV \cdot s^{-1}$ in 0.1 mol/L $HClO_4$ until a stable CV curve was obtained. The CV curve was recorded at room temperature in a N_2-saturated 0.1 M

$HClO_4$ solution at a scanning rate of 50 mV/s in the potential range of 0.08−1.78 V_{RHE}. The specific ECSAs were calculated from the charges associated with the desorption of hydrogen in the region of 0.08-0.45 V_{RHE}.

To compare the ORR activities of the as-prepared Au-Pt catalysts and commercial Pt/C catalysts, the Au-Pt bimetallic samples were loaded on a carbon support with a metal loading content of 20% based on the total mass of Au and Pt. After drying, 6.0 mg of the carbon-supported Au-Pt catalysts were dispersed in 4.0 mL of ethanol and 80 μL of 5% Nafion under ultrasonication for 20 min. 20 μL of the suspension were deposited on a pre-cleaned glassy carbon rotating disk electrode (RDE) with a geometric area of 0.196 cm^2 and dried in an oven at 50 °C.

The glassy carbon electrode loaded with the as-prepared catalysts was electrochemical cleaned by continuous potential cycling between -0.08 and 1.10 V at 50 $mV{\cdot}s^{-1}$ in 0.1 mol/L $HClO_4$ until a stable CV curve was obtained. The CV curve was recorded at room temperature in a N_2-saturated 0.1 M $HClO_4$ solution at a scanning rate of 50 mV/s in the potential range of 0.08−1.1 V_{RHE}. The specific ECSAs were calculated from the charges associated with the desorption of hydrogen in the region of 0.08-0.45 V_{RHE} after double-layer correction by taking a value of 210 $\mu C/cm^2$ for the desorption of a monolayer of hydrogen from Pt surfaces. The ORR activity was measured in the potential range of 0.08-1.1 V_{RHE} in an O_2-saturated 0.1 M aqueous $HClO_4$ solution using the RDE method at room temperature with a scanning rate of 10 mV/s (RDE rotating rate of 1,600 rpm). We calculated the kinetic currents from the ORR polarization curves by following the Koutecky-Levich equation: $1/i = 1/ i_k + 1/i_d$ (where i_k is the kinetic current and i_d is the diffusion-limiting current). The iR compensation was not used for the ORR measurement. For the accelerated durability test, the CVs and ORR polarization curves were measured after sweeping 5,000 and 10,000 cycles between 0.6 and 1.1 VRHE at a rate of 0.1 V/s in an O_2-saturated aqueous HClO4 solution (0.1 M) at room temperature.

Electrochemical oxidation of formic acid. A glassy carbon electrode (diameter of 3 mm) was carefully polished and washed before every test. 10 μl of the ethanol suspensions (0.2 mg_{Pt}/mL) of the as-prepared Au-Pt NCs or commercial Pt/C catalyst were dripped onto

the surface of the glassy carbon electrode and dried at 50 ^{o}C in an oven. The glassy carbon electrode loaded with the as-prepared Au-Pt NCs or Pt/C catalyst was electrochemical cleaned by continuous potential cycling between 0.08 and 1.10 V at 100 $mV{\cdot}s^{-1}$ in 0.1 M $HClO_4$ until a stable cyclic voltammogram curve was obtained. Electro-oxidation of formic acid was conducted in a solution containing 0.50 M $HClO_4$ and 0.50 M HCOOH at a sweeping rate of 50 $mV{\cdot}s^{-1}$. The electrochemical reactivity and electrochemically active surface area of the catalysts were determined by the area of the hydrogen desorption peaks in the cyclic voltammetry measurement performed in 0.5 M $HClO_4$ electrolyte at a scan rate of 50 $mV{\cdot}s^{-1}$ (25 ^{o}C). Before electrochemical measurements, the electrolyte was purged with N_2 for 0.5 h to remove the oxygen in the electrolyte.

Instrumentation. The morphology and structure of the products were characterized by scanning electron microscopy (SEM, S-4800), transmission electron microscopy (TEM, JEM-2100) and X-ray powder diffraction (XRD, D/MAX-2500 with Cu-Kα radiation). The HAADF-STEM-EDS was performed on the JEM-2100 microscope operated at 200 kV.

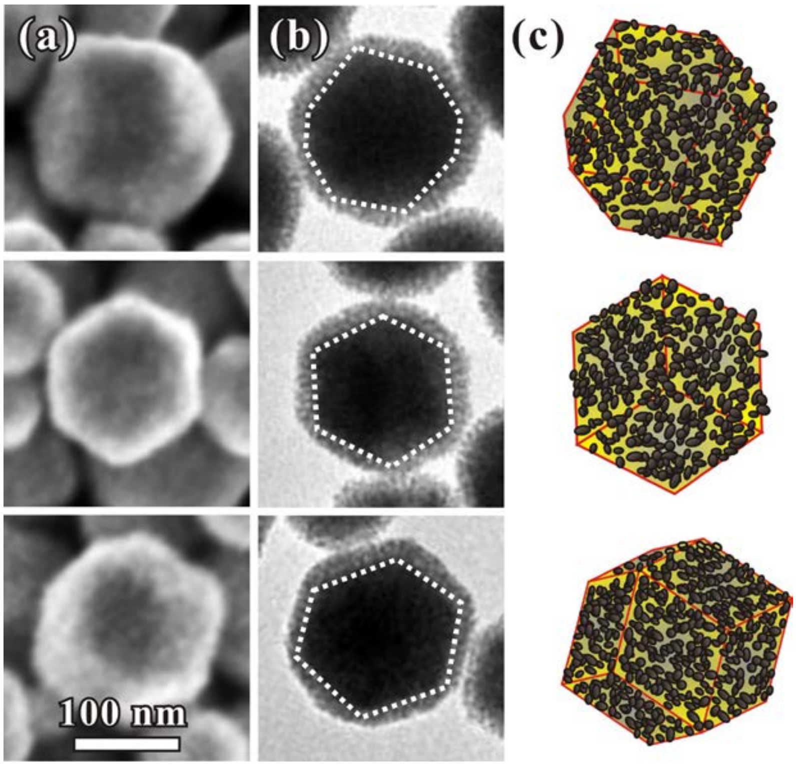

Figure S1. (a, b) A series of high-magnification SEM and TEM images of the as-prepared Au-Pt NCs viewing from different directions. (c) The corresponding models of the rhombic dodecahedral model with branches projected from different orientations, which match the outlines of the obtained NCs well.

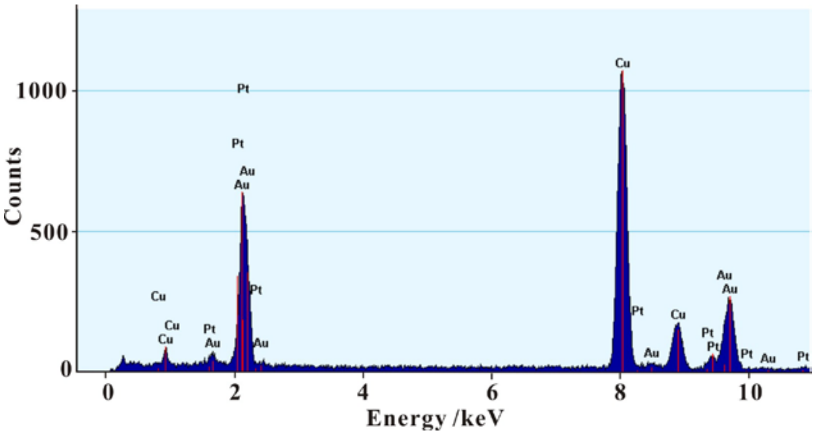

Figure S2. EDS spectrum of the porous rhombus deodecahedral Au-Pt nanocrystals that were prepared using the standard procedure.

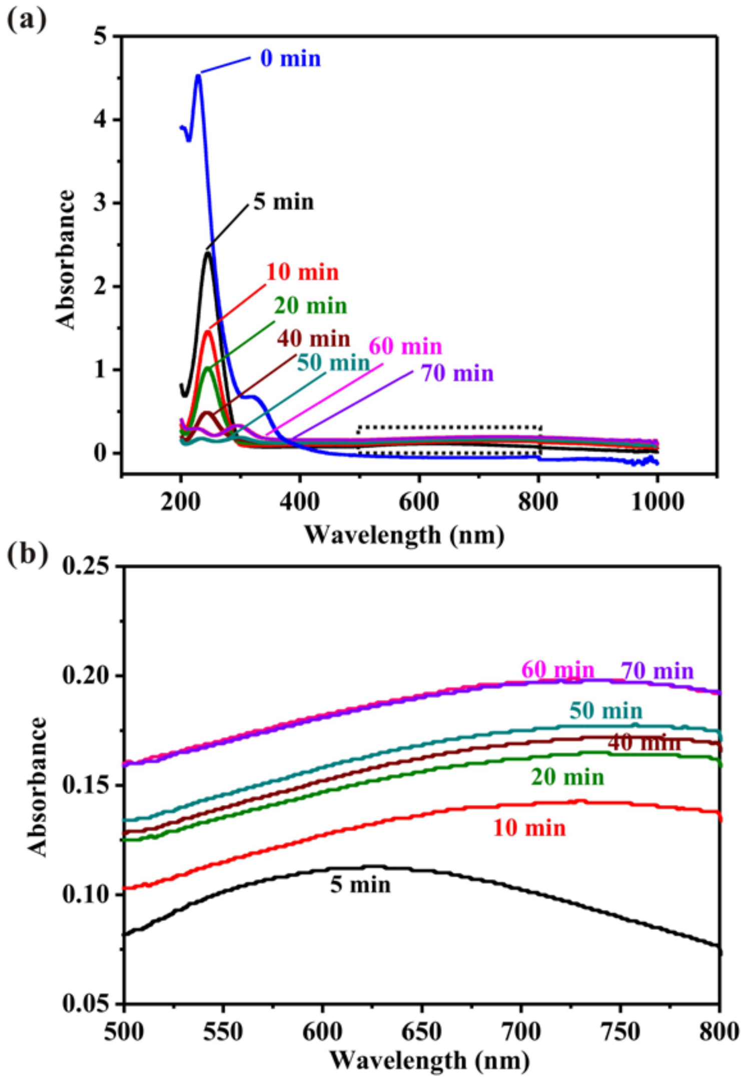

Figure S3. (a) UV-vis spectra taken from reaction solutions at different stages of the synthesis, which was performed under the typical procedure. (b) The enlarged UV-vis spectra from the region boxed in a.

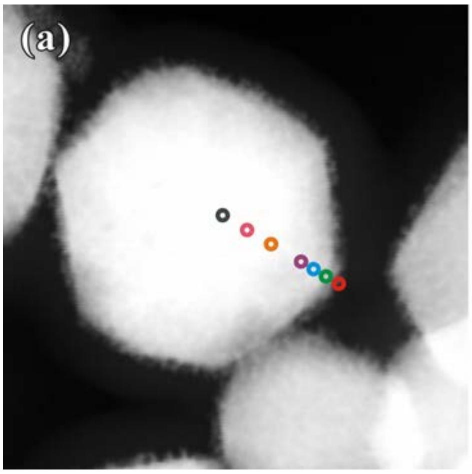

(b)

Position	Pt Atomic %
○ (red)	100.0
○ (green)	97.2
○ (blue)	64.2
○ (purple)	42.5
○ (orange)	25.4
○ (pink)	20.8
○ (black)	16.1

Figure S4. (a) The HAADF-STEM image of one individual NC. (b) The Pt atomic ratios on different positions of the particle, which were confirmed by EDS analysis.

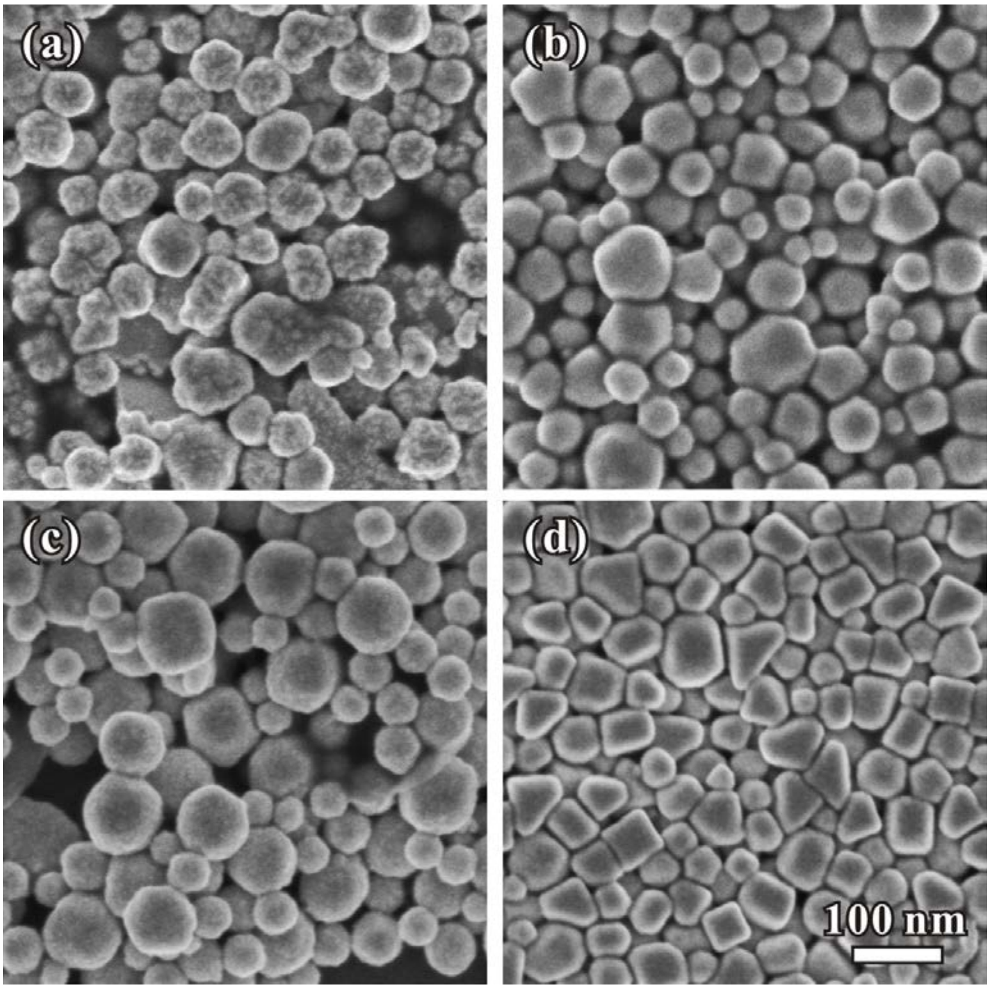

Figure S5. (a, b) TEM images of Au-Pt bimetallic NCs that were prepared using the standard procedure, except for reacting at a) room temperature and b) 90 ^oC. (c, d) TEM images of Au-Pt products that were prepared using the standard procedure, except for the use of c) CTAC and d) OTAB, as the surfactant in the same molar amount as the OTAC.

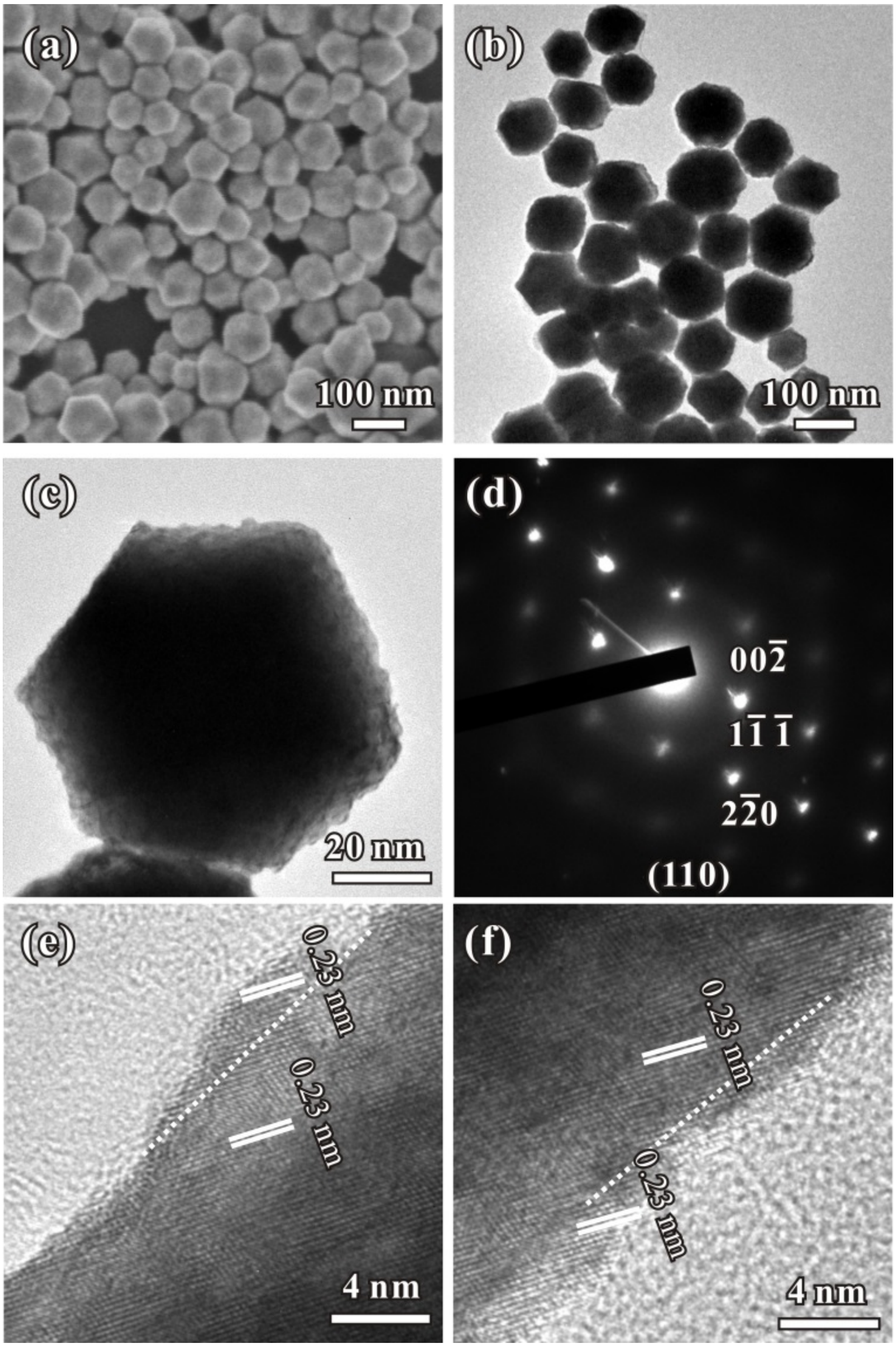

Figure S6. (a, b) The SEM and TEM image of the porous rhombus dodecahedral $Au_{75}Pt_{25}$ bimetallic NCs. (c, d) The TEM image and corresponding SAED pattern of one individual $Au_{75}Pt_{25}$ bimetallic nanocrystal. (e, f) HR-TEM images of two regions for one same nanocrystal.

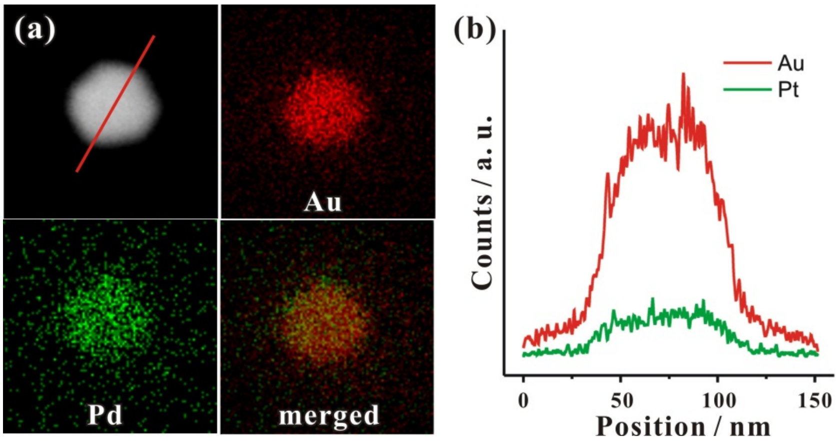

Figure S7. (a) HAADF-STEM image and corresponding HAADF-STEM-EDS elemental mapping of one individual $Au_{75}Pt_{25}$ bimetallic nanocrystal. (b) HAADF-STEM-EDS cross-sectional compositional line profile as marked in the STEM image.

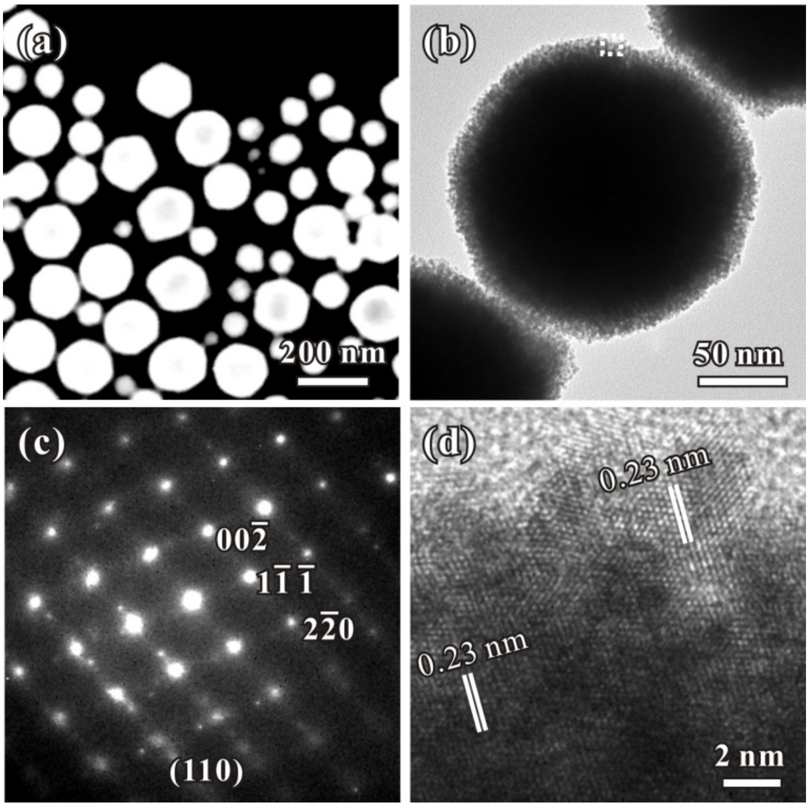

Figure S8. (a) The STEM image of the $Au_{50}Pt_{50}$ bimetallic NCs. (b, c) The TEM image and corresponding SAED pattern of one individual $Au_{50}Pt_{50}$ bimetallic nanocrystal. (d) HR-TEM images of the region marked in (b).

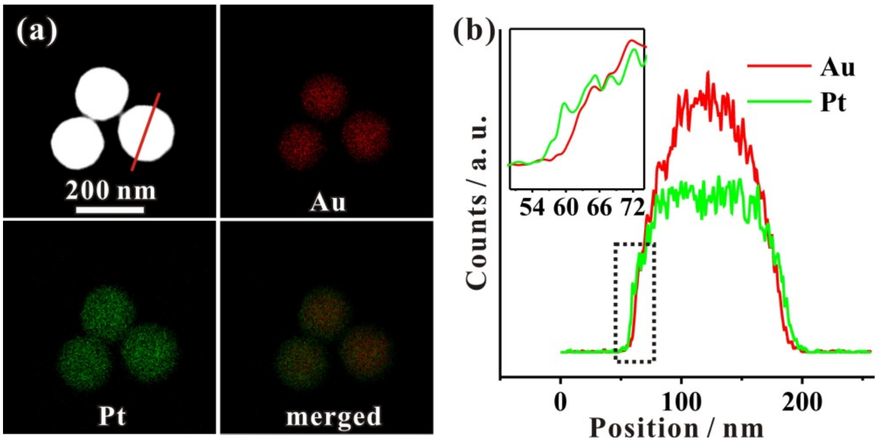

Figure S9. (a) HAADF-STEM image and corresponding HAADF-STEM-EDS elemental mapping of one individual $Au_{50}Pt_{50}$ bimetallic dodecahedron. (b) HAADF-STEM-EDS cross-sectional compositional line profile as marked in the STEM image.

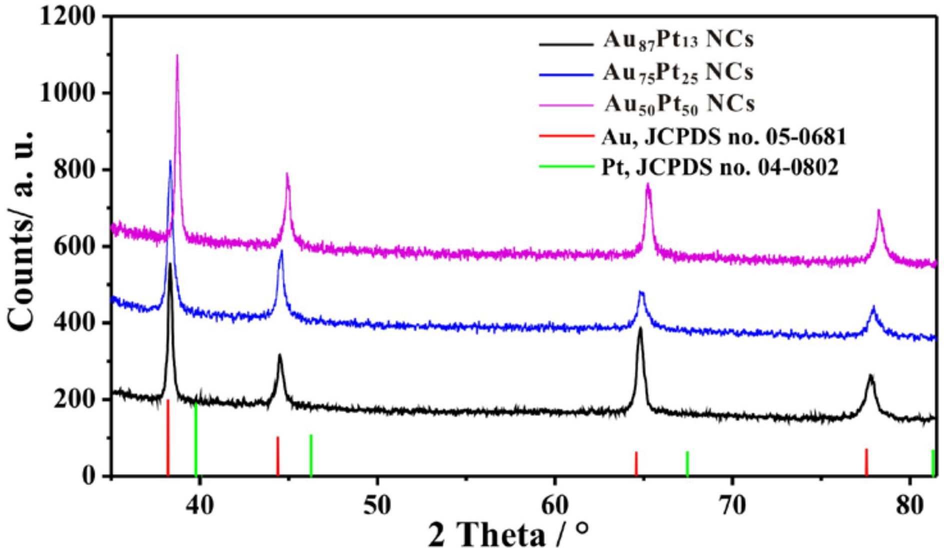

Figure S10. XRD patterns of Au-Pt bimetallic NCs which were prepared using the standard procedure, except for the variation in the amount of K_2PtCl_4 (1mM): 0.5 mL (black), 1.0 mL (blue), and 3.0 mL (pink), respectively.

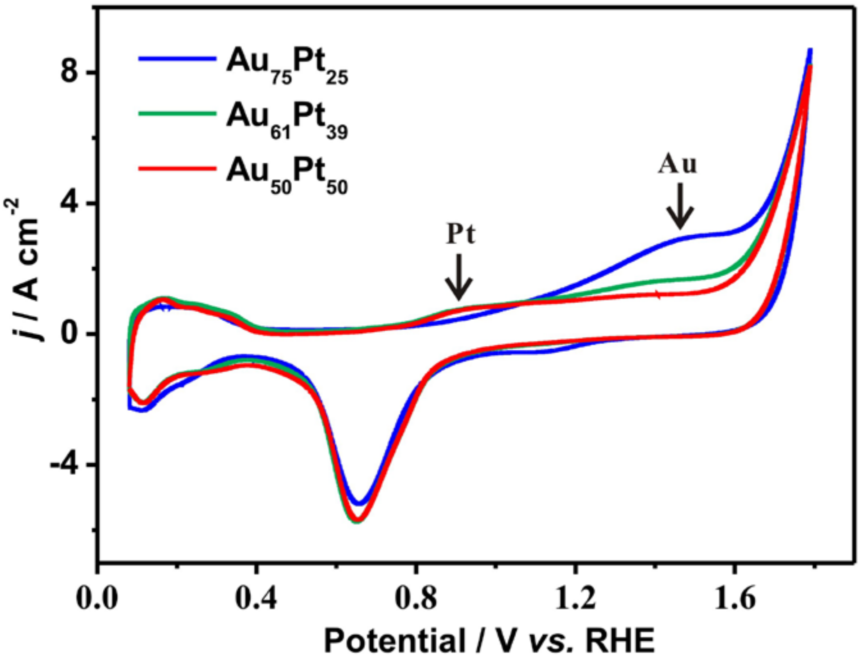

Figure S11. The CV curves of $Au_{75}Pt_{25}$, $Au_{61}Pt_{39}$, $Au_{50}Pt_{50}$ catalysts in the potential region from 0.08 to 1.78 V.

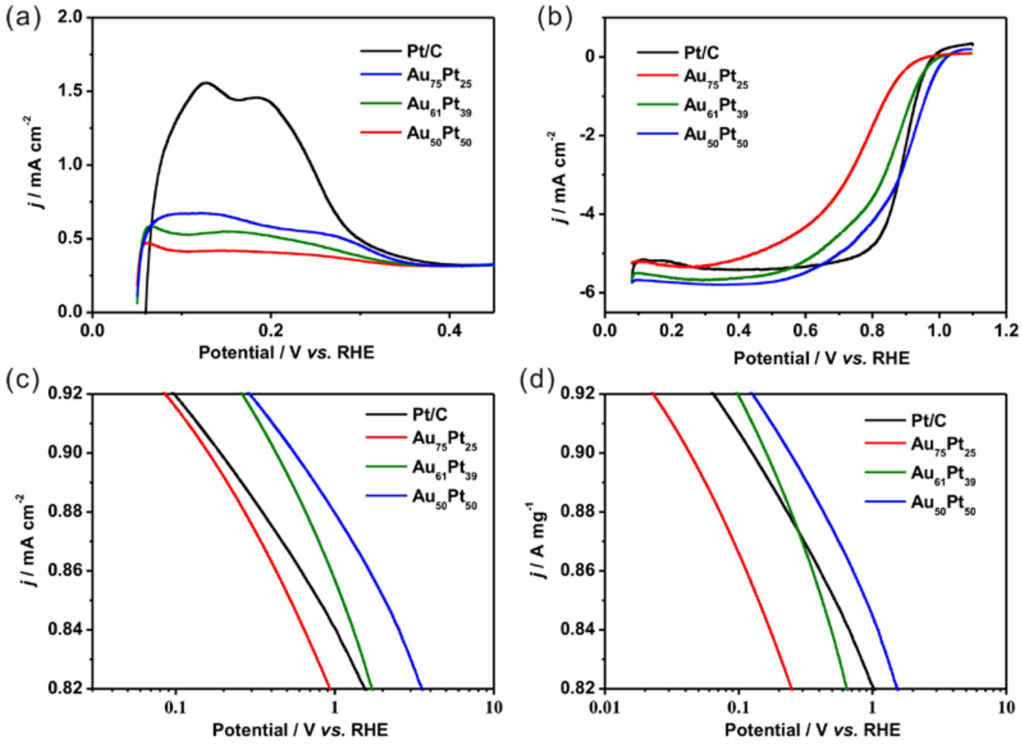

Figure S12. (a) The H desorption peaks in the potential region from 0.08 to 0.46 V. (b) ORR polarization curves of the $Au_{75}Pt_{25}$, $Au_{61}Pt_{39}$ and $Au_{50}Pt_{50}$ catalysts in comparison with a commercial Pt/C catalyst. The current densities were normalized to the geometric area of the RDE (0.196 cm^2). (c) Specific and (d) mass activities given as kinetic current densities (j_k) normalized against the ECSAs of the catalysts and the mass of Pt, respectively.

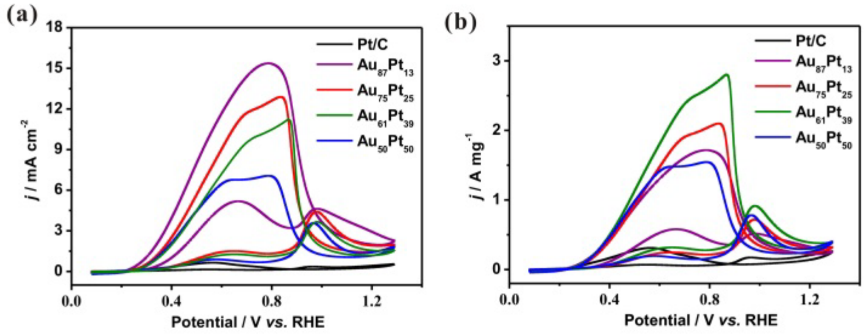

Figure S13. Cyclic voltammograms of the four Au-Pt bimetallic NCs with different thickness of Pt dendritic branches, together with that of commercial Pt/C catalyst. The curve was recorded at room temperature in an aqueous solution containing 0.5 M HCOOH and 0.5 M $HClO_4$ at a sweeping rate of 50 mV·s^{-1}. The current was normalized to the corresponding (a) ECSAs and (b) mass of Pt, respectively.
